# Comparison of the NHANES dietary screener questionnaire to the Automated Self-Administered 24-Hour Recall for Children in the Healthy Communities Study

**DOI:** 10.1186/s12937-018-0415-1

**Published:** 2018-11-27

**Authors:** Sridharshi Chintha Hewawitharana, Frances Elizabeth Thompson, Catherine M. Loria, Warren Strauss, Jyothi Nagaraja, Lorrene Ritchie, Karen Lucy Webb

**Affiliations:** 1 Nutrition Policy Institute, University of California, Agriculture and Natural Resources Division, 2115 Milvia Street, Third floor, Berkeley, CA 94704 USA; 2 Risk Factor Assessment Branch, Epidemiology and Genomics Research Program, Division of Cancer Control and Population Sciences, National Cancer Institute, Rm. 4E124, 9609 Medical Center Drive, MSC 9763, Bethesda, MD 20892 USA; 3 Division of Cardiovascular Sciences, National Heart, Lung, and Blood Institute, 6701 Rockledge Drive, Suite 10018, Bethesda, MD 20892 USA; 4 Battelle, 505 King Ave, Columbus, OH 43201 USA

**Keywords:** ASA24-kids, FFQ, Dietary screener, DSQ, Concurrent validity

## Abstract

**Background:**

A dietary screener questionnaire (DSQ) was used to assess dietary outcomes among children in the Healthy Communities Study (HCS), a study of the relationships between programs and policies to prevent child obesity and child diet, physical activity and weight outcomes.

**Methods:**

To compare dietary intake estimates derived from the DSQ against those from the Automated Self-Administered 24-Hour Recalls for Children (ASA24-Kids) among children, a measurement error model, using structural equation modelling, was utilized to estimate slopes, deattenuated correlation coefficients, and attenuation factors by age and sex, ethnicity, and BMI status.

**Participants/setting:**

A randomly selected sub-sample of HCS participants aged 4–15 years in 130 communities throughout the U.S. who completed the DSQ and up to two ASA24-Kids recalls (*n* = 656;13% of HCS participants).

**Results:**

For most nutrient/foods examined, the DSQ yielded larger mean intake estimates than the ASA24-Kids, and agreement between the two measures varied by food/nutrient, age and sex, ethnicity, and BMI category. Deattenuated correlation coefficients of 0.4 or greater were observed for added sugars from SSBs (0.54), fruits and vegetables (0.40), and dairy foods (0.50). Lower deattenuated correlation coefficients were seen for total added sugars (0.37), whole grains (0.34), and fiber (0.34). Attenuation factors were most severe for total added sugars intake among overweight children, and for several other dietary outcomes among children aged 9–11 years.

**Conclusions:**

The DSQ was found to be a tool with acceptable agreement with the ASA24-Kids for measuring multiple dietary outcomes of interest in the HCS, although there may be potential due to measurement error to underestimate results (bias towards the null). In future studies, measurement error modelling and regression calibration may be possible solutions to correct for bias due to measurement error in most food/nutrient intake estimates from the DSQ when used among children.

**Electronic supplementary material:**

The online version of this article (10.1186/s12937-018-0415-1) contains supplementary material, which is available to authorized users.

## Background

Assessing dietary intakes can be done in multiple ways, but each method has advantages and disadvantages [1]. For example, the use of recovery biomarkers allows intakes to be measured without relying on self-report but limits outcomes to those for which there are known recovery biomarkers. In contrast, assessing diet through methods reliant on self-report such as 24-hour recalls, food frequency questionnaires, and dietary screeners allow for the examination of a greater number of food groups and nutrients of interest, but also contain varying degrees of measurement error due to factors such as recall error and social desirability bias. Measurement error not only varies by dietary assessment tool, but also by dietary intake and study population.

In addition to the expected amount of measurement error, other concerns such as feasibility, cost, and participant burden play a role in determining the most appropriate tool to use in a study. Researchers must make tradeoffs to obtain the most accurate measures for the study population and resources available, and accept the type and magnitude of measurement error associated with the method of choice. For example, 24-hour recalls tend to have less measurement error than food frequency questionnaires and dietary screeners, but are more resource-intensive and require more from participants to complete. Regardless of the dietary assessment method used, it is crucial for researchers to be aware of the nature, direction and magnitude of measurement error so as to interpret results appropriately.

This paper aims to describe the measurement error present in intake estimates of added sugars, added sugars from sugar-sweetened beverages (SSBs), fruits and vegetables, whole grains, fiber, and dairy derived from the NHANES dietary screener questionnaire (DSQ) among children 4–15 years of age. In this study, we have evaluated agreement with intake estimates derived from the Automated Self-Administered 24-Hour Recall for Children (ASA24-Kids), using data from the Healthy Communities Study (HCS) (see Additional file 1).

The HCS aimed to assess associations between community programs and policies to prevent childhood obesity and outcomes including nutrition and physical activity behaviors and BMI, in a large, diverse sample of children from 130 communities across the United States. To accomplish this, a measure of dietary intake that could easily be administered by a large number of field data collectors for a large sample of children across the country was required. Dietary assessments were made on the entire HCS sample of over 5000 children, using a modified version of the DSQ [2] that was developed by the National Cancer Institute (NCI) and used with children and adults in the National Health and Nutrition Examination Survey (NHANES) [3].

This short food frequency questionnaire-based method was selected for use in the HCS because it assessed dietary factors related to obesity risk and because it was feasible to administer to a large study population in a short time by field interviewers who had no special nutrition training [2, 4]. It also allowed for comparison to national data using the same method and enabled the calculation of quantitative intake estimates from reported frequencies [2]. Previously, DSQ intake estimates for foods such as fruits and vegetables have been shown to correlate well with estimates from interviewer-administered 24-hour recalls among adults [5]. However, as a brief dietary screener, the DSQ relies on complex cognitive tasks to calculate usual frequency of intake of various foods and is thus more challenging and potentially less accurate when used with children [1].

While the DSQ was the most feasible dietary assessment tool to be used in the HCS, information was lacking on the measurement error associated with intake estimates in relation to a more accurate dietary assessment method, especially among children. Dietary assessments on a subset of the HCS sample were therefore made using a 24-hour dietary recall to describe and quantify the measurement error present in DSQ intake estimates. The ASA24-Kids recall was selected as the reference method with which to compare the DSQ in the HCS. The ASA24-Kids was the most feasible, affordable comparison method because it is a standardized, automated online interview and does not require specialist trained nutrition researchers for data collection. While past studies have shown that some children at younger ages find it difficult to accurately report their own intakes using the ASA24-Kids, other studies have shown that among adults, the ASA24 yields results comparable to that of interviewer-administered 24-hour recalls [6–8]. Two recalls were collected from each of the sub sample respondents to adjust for in-person variation [2].

## Methods

### Study population

Sampling, measures, and methods used in the HCS have been described in detail elsewhere [2, 9–14]. In brief, HCS study participants (*n* = 5138) were recruited from a large, stratified sample of 130 high school catchments areas, or “communities”, across the U.S. [9]. By design, the HCS oversampled lower income and minority communities and was not a representative sample of US children. Within each community, up to two elementary and two middle schools, were selected as the recruitment base for household/child participants [9]. Up to 81 children in grades K-8 were selected per community to complete the standard set of assessments, including the DSQ [9]. Letters requesting participation and study interest forms were sent home and those households returning an interest form were scheduled for home visits [9]. A random subsample of about 14% of the households consenting to participate in the HCS were selected for the enhanced protocol which included more detailed measures of dietary intake, such as the ASA24-Kids recall, in addition to the standard set of assessments. The study population for this paper comprises the sub-sample of the HCS participants with a completed DSQ who completed at least one ASA24-Kids recall.

The research protocol was approved by OMB and the Battelle Memorial Institute IRB. Parents provided written informed consent for child participation [2].

### Dietary assessment measures

The 27-item DSQ was adapted from the version administered to those aged 2–69 years of age in NHANES 2009–2010 [2]. An expert committee was convened by the lead investigators to advise on dietary assessment methods. Modifications of the DSQ for the HCS were minimal. In an attempt to reduce time required for home visits, DSQ items were prioritized and variables selected that have shown moderate to strong evidence of a relationship to obesity, including sugar sweetened beverages, fruits and vegetables among others. Questions about meat intake were excluded and a question about the frequency of chip and cracker consumption was added, although the latter was used only to assess the frequency of intake of discretionary foods, and, as such, was not used for quantitative intake estimates analyzed in this paper. [2]. While fast food intake was not assessed by the DSQ, a question about frequency of intake was included. Participants or their proxy reported the frequency of consuming selected foods and food groups over the past 30 days in number of times per day, week, or month. Quantitative estimates for intakes of each of the food groups and nutrients were calculated from the DSQ using publically available scoring algorithms derived from NHANES 2009–2010 data. To calculate scores, the reported frequencies of consumption were standardized to daily frequencies, to which age and sex specific median quantities of selected food groups were applied, and calibration equations were computed, derived from regressing DSQ with 24-hour recalls [15].

The ASA24-Kids is a 24-hour recall web-based program which was developed by the NCI for use with children and is available in English and Spanish [16, 17]. It is based on the Automated Multiple Pass Method used in interviewer administered recalls for NHANES, and adapted for online administration [18–20]. The ASA24-Kids consists of an ‘interview’ conducted by an animated character that queries the respondent in stages about all foods and beverages consumed in the previous 24 hours. During each stage or “pass”, the respondent is probed for forgotten foods and beverages; time and eating occasion; and amounts consumed using photographs of graduated serving sizes [21]. The ASA24-Kids (2012 and 2014 versions) utilized the Food and Nutrient Database for Dietary Studies 4.1 as the basis for calculating intakes of nutrients, and food groups. “MyPyramid” serving equivalents were calculated within the ASA24-Kids program using the MyPyramid Equivalents Database 2.0 [16].

### Data collection

Dietary data was collected during home visits, with the parent/guardian and child present. The DSQ and ASA24-Kids recalls were collected in English or Spanish, according to participant preference [2]. For both dietary assessments, the primary respondent was selected based on the child’s age as follows: the parent/adult proxy with child assistance for children 4–8 years; the child with parent/adult proxy assistance for children 9–11 years; the child with parent/adult proxy assistance only if needed for children 12–15 years [2].

Trained field data collectors (FDCs) administered the DSQ, giving a neutral, scripted introduction and directions to the parent or adult caregiver and child concerning who should respond and how to obtain clarification from parents when the child was the primary respondent [2]. The FDC entered respondents’ answers into a pre-programmed electronic tablet [2]. Out of range responses prompted a neutral scripted probe by the interviewer to verify or correct responses [2].

Respondents in the enhanced protocol sub-sample completed the ASA24-Kids on an HCS tablet computer supplied by FDCs during home visits [2]. Study participants were asked to complete two recalls. The first was collected on the same day as the interviewer administration of the DSQ, and the second recall occurred at a follow up home visit approximately 7–10 days later. The FDCs’ role in administering the ASA24-Kids was restricted to logging onto the program, troubleshooting, and providing minimal assistance [2]. To avoid potential reactivity, respondents were unaware that they would be completing 24-hour recalls prior to the home visit [2].

Participant sex, age, race, and ethnicity were determined by survey responses and BMI was calculated from measured height and weight recorded by trained FDCs.

### Training and quality assurance

FDCs were trained to administer the DSQ and oversee completion of the ASA24-Kids [2]. To assure adherence to the protocol, field supervisors and senior research staff conducted quality assurance checks of FDCs on selected home visits [2].

Data quality was monitored monthly for missing data, irregularities in administration time, extreme values at the low and high ends of the distribution, and percentage of ‘don’t know’ and ‘refusal’ responses for the DSQ [2]. Data obtained from the ASA24-Kids was also reviewed monthly, for response rates, incompletes, documentation about reasons for missing or incomplete recalls, comparison with age and sex specific values from NHANES 2010, and review of outliers [2]. FDCs with incomplete or other data issues participated in refresher training and were monitored subsequently [2].

### Statistical analyses

Box-Cox transformations were used to find the best transformation to achieve normal distributions for each food group or nutrient intake on the completed recalls (*n* = 656). Transformed data were reviewed, and extreme outliers were excluded from analysis for those particular nutrients or food groups. Extreme outliers were defined as those with nutrients or food groups at least two times the interquartile range lower or higher than the lower and upper bounds of the interquartile range (25th and 75th percentiles). For each intake of interest, full records were excluded if intake estimates from both recalls were missing or identified as extreme outliers, or if the DSQ intake estimate was missing (*n* = 7 for total added sugars; *n* = 4 for added sugars from SSBs; *n* = 9 for fruits and vegetables; n = 4 for whole grains; *n* = 18 for fiber; and *n* = 3 for dairy). All statistical analyses were conducted using SAS (version 9.4, 2013, SAS Institute Inc.).

Mean differences between the DSQ and ASA24-Kids intake estimates (mean of two recall days if available) were assessed by paired t-tests. A measurement error model using structural equation modelling, similar to that described by Freedman et al. [22], was applied to estimate relationships between estimated usual intake, as derived from the recalls, and the DSQ intake estimates. ASA24-Kids intake estimates were modelled as true usual intake plus random error. The modelling removed intra-individual variation to estimate true usual intake, with the assumption that the recalls were unbiased at the individual level and only contained intra-individual random error [23]. DSQ intakes were then modelled as a function of the estimated true usual intake plus error, both random and systematic. This model was fit by age group and sex, ethnicity, and BMI category to the DSQ and ASA24-Kids recall data and yielded estimates of measurement error characteristics of the DSQ. Models were not adjusted for covariates because we sought to assess the direct relationship between these two measurement methods and how these measures relate to a theoretical estimate of true intake using a structural equation modelling approach; the addition of covariates to the model would have distorted these relationships. The slope (β_1_) in the regression of reported intake from the DSQ on estimated true usual intake, using the recalls, as well as the deattenuated correlation coefficient between reported and estimated usual intakes (R), and the attenuation factor (λ = R^2^/ β_1_) were then estimated using full information maximum likelihood estimation.

If the DSQ were unbiased, the regression of reported intake on true usual intake would have a slope of one and an intercept of zero [23]. Deattenuated correlations assess the agreement between reported intake and estimated usual intake, adjusted for intra-individual variation. The attenuation factor indicates the degree to which the observed log relative risk between the observed exposure of a dietary intake derived from the DSQ and an outcome of interest is biased due to measurement error [23]. Attenuation factors typically range from 0 to 1, indicating bias towards the null (underestimation of true association), with values closer to zero indicating more severe attenuation [23]. Attenuation factors greater than one indicate bias away from the null (overestimation of true association) [23].

## Results

Of the 5138 participants in the HCS, 5123 completed a DSQ, and 712 (14%) were randomly selected for the enhanced protocol. Of those, 656 participants (92.1%) completed one recall and 551 participants (77.4%) completed both recalls. The sample size for the analysis of each intake variable ranged from 638 to 653. The sample comprised approximately 50% females (Table 1). A higher proportion of participants were 4–8 years old, than 9–11 year olds, or 12–15 year olds. Over half of the participants were white, non-Hispanic, were of normal weight, and had a family income of less than $35,000 per year. Approximately 40% had parents who had attained a high school education or less. Overall there were no statistically significant differences in sociodemographic characteristics between the enhanced protocol subsample and the entire HCS sample.

**Table 1:** Comparison of sociodemographics of Enhanced Protocol sub-sample and Total sample, Healthy Communities Study, USA, 2013–2015

	Number of Participants (*n* (%))		*P*-value^a^
	Enhanced Protocol Sub-Sample (*n* = 656)	Total HCS Sample (*n* = 5138)	
1. Demographics			
Sex/Age Category			0.83
Male	323 (49.2)	2524 (49.1)	
4-8y	136 (20.7)	1099 (21.4)	
9-11y	100 (15.2)	815 (15.9)	
12-15y	87 (13.3)	610 (11.9)	
Female	333 (50.8)	2614 (50.9)	
4-8y	132 (20.1)	1097 (21.4)	
9-11y	113 (17.2)	824 (16.0)	
12-15y	88 (13.4)	693 (13.5)	
BMI Category			0.26
Normal weight	366 (55.8)	3022 (58.8)	
Overweight	105 (16.0)	789 (15.4)	
Obese	180 (27.4)	1264 (24.6)	
Missing	5 (0.8)	63 (1.2)	
Race			0.35
White	360 (54.9)	2924 (56.9)	
African American	136 (20.7)	960 (18.7)	
American Indian/Alaskan Native	7 (1.1)	59 (1.2)	
Native Hawaiian/Pacific Islander	1 (0.2)	8 (0.2)	
Asian	14 (2.1)	167 (3.3)	
Multiracial	45 (6.9)	227 (4.4)	
Missing	93 (14.2)	793 (15.4)	
Ethnicity			0.26
Hispanic	265 (40.4)	2225 (43.3)	
Non-Hispanic	375 (57.2)	2767 (53.9)	
Missing	16 (2.4)	146 (2.9)	
2. By socioeconomic status			
Income per year			0.57
Less than $20,000	168 (25.6)	1261 (24.5)	
$20,000–$35,000	144 (22.0)	1109 (21.6)	
$35,000–$50,000	88 (13.4)	602 (11.7)	
$50,000–$75,000	59 (9.0)	517 (10.1)	
$75,000–$100,000	54 (8.2)	383 (7.5)	
Greater than or equal to $100,000	96 (14.6)	840 (16.4)	
Missing	47 (7.2)	426 (8.3)	
Maximum parent educational attainment^b^			0.39
Did not attend high school	41 (6.3)	434 (8.5)	
Some high school, no diploma	95 (14.5)	713 (13.9)	
High school diploma, GED or equivalent	136 (20.7)	979 (19.1)	
Some college, no degree	91 (13.9)	646 (12.6)	
Associate or bachelor degree	175 (26.7)	1347 (26.2)	
Masters, doctoral, or professional degree	98 (14.9)	844 (16.4)	
Missing	20 (3.1)	175 (3.4)	

^a^Differences in categorical variables assessed via Chi-squared test

^b^Highest level of education attained by parent

Relative to the ASA24-Kids recalls, the mean intake estimates from the DSQ for all nutrients/foods were significantly higher for the total sample and for nearly all subgroups (Table 2). Differences in means between the two measures were not significantly different for girls aged 9–11 years on added sugars from SSBs, for girls aged 9–15 years on fruits and vegetables, and for girls aged 4–8 years for whole grains.

**Table 2:** Differences between children’s dietary intakes measured via DSQ and ASA24-Kids, Healthy Communities Study, USA, 2013–2015

	DSQ		ASA24-Kids		Mean difference between dietary intakes as measured by DSQ and ASA24-Kids			
Food Group/ Nutrient	Mean ± SD	Median	Mean ± SD	Median	Mean Difference^a^	Percent Change^b^	Paired t-test test statistic	*P*-value
1. Total added sugars (tsp/day)								
All (*n* = 649)	19.0 ± 7.4	17.2	12.2 ± 8.3	10.7	− 6.8	− 55.7%	− 17.8	< 0.01
Sex and age								
Male (*n* = 321)	20.1 ± 7.8	18.2	13.0 ± 8.7	11.3	− 7.1	− 54.6%	− 12.3	< 0.01
4-8y (*n* = 136)	18.3 ± 5.0	17.1	11.0 ± 6.7	9.4	− 7.2	− 66.4%	− 11.0	< 0.01
9-11y (*n* = 98)	19.6 ± 8.3	17.6	13.8 ± 8.1	13.3	− 5.7	− 42.0%	− 5.0	< 0.01
12-15y (*n* = 87)	23.5 ± 9.7	20.8	15.2 ± 11.3	12.8	− 8.3	− 54.6%	− 6.3	< 0.01
Female (*n* = 328)	17.9 ± 6.8	16.3	11.3 ± 7.8	10.3	− 6.6	− 58.4%	− 12.8	< 0.01
4-8y (*n* = 131)	16.0 ± 3.9	15.1	10.6 ± 6.3	10.0	− 5.5	− 50.9%	− 9.2	< 0.01
9-11y (*n* = 112)	18.2 ± 5.2	17.2	12.8 ± 9.1	10.9	− 5.5	− 42.2%	− 5.6	< 0.01
12-15y (*n* = 85)	20.3 ± 10.4	17.5	10.6 ± 7.9	9.8	−9.7	− 91.5%	−8.6	< 0.01
BMI category^c^								
Normal weight (*n* = 363)	18.9 ± 7.6	17.1	12.0 ± 8.1	10.6	− 6.9	− 57.5%	− 13.5	< 0.01
Overweight (*n* = 104)	18.5 ± 5.9	17.2	12.0 ± 8.8	10.9	− 6.5	− 54.2%	− 6.8	< 0.01
Obese (*n* = 177)	19.4 ± 7.8	17.5	12.7 ± 8.7	10.9	− 6.7	− 52.8%	− 9.0	< 0.01
Race								
White (*n* = 357)	18.3 ± 6.1	17.0	12.8 ± 8.3	11.1	−5.5	− 43.0%	− 11.8	< 0.01
African American (*n* = 135)	22.9 ± 11.1	19.8	12.8 ± 9.2	11.0	−10.1	−78.9%	− 8.7	< 0.01
Other (*n* = 66)	17.1 ± 4.2	16.3	11.5 ± 6.7	10.7	−5.6	− 48.7%	− 6.3	< 0.01
Ethnicity								
Hispanic (*n* = 262)	18.4 ± 5.8	17.2	11.1 ± 7.5	10.2	−7.4	− 65.8%	−15.1	< 0.01
Non-Hispanic (*n* = 371)	19.2 ± 7.9	17.1	13.0 ± 8.8	11.1	−6.2	− 47.7%	−11.3	< 0.01
2. Added sugars from sugar sweetened beverages (tsp/day)								
All (*n* = 652)	7.1 ± 4.8	5.6	4.9 ± 5.4	3.6	− 2.1	− 44.9%	− 9.7	< 0.01
Sex and age								
Male (*n* = 321)	7.4 ± 3.8	6.3	5.4 ± 5.9	4.0	−1.9	− 37.0%	−6.9	< 0.01
4-8y (*n* = 136)	6.1 ± 2.7	5.2	4.0 ± 3.6	3.4	− 2.1	−52.5%	−6.5	< 0.01
9-11y (*n* = 98)	6.6 ± 3.2	5.6	5.6 ± 5.6	3.8	−1.0	− 17.9%	− 2.1	0.04
12-15y (*n* = 87)	10.3 ± 4.4	8.8	7.5 ± 8.2	5.6	− 2.8	− 37.3%	− 3.9	< 0.01
Female (*n* = 331)	6.8 ± 5.5	4.9	4.4 ± 4.9	3.2	−2.3	−54.5%	−6.9	< 0.01
4-8y (*n* = 132)	5.3 ± 2.7	4.3	3.8 ± 3.7	2.8	−1.5	− 39.5%	−4.5	< 0.01
9-11y (*n* = 112)	6.0 ± 2.9	4.7	5.1 ± 5.3	4.0	− 0.8	−17.6%	− 1.6	0.11
12-15y (*n* = 87)	10.0 ± 8.9	6.8	4.5 ± 5.8	3.2	−5.5	− 122.2%	−6.2	< 0.01
BMI category^c^								
Normal weight (*n* = 365)	6.8 ± 4.4	5.4	4.8 ± 5.6	3.3	− 2.1	−41.7%	−6.7	< 0.01
Overweight (*n* = 104)	7.0 ± 3.7	5.8	4.4 ± 4.5	3.6	− 2.5	− 59.1%	− 5.5	< 0.01
Obese (*n* = 178)	7.7 ± 5.9	5.8	5.7 ± 5.6	4.5	− 2.1	− 35.1%	− 4.8	< 0.01
Race								
White (*n* = 358)	6.6 ± 4.2	5.4	5.0 ± 5.6	3.5	− 1.6	− 32.0%	− 5.7	< 0.01
African American (*n* = 136)	9.3 ± 6.6	7.4	5.4 ± 5.3	4.3	− 3.9	− 72.2%	− 6.2	< 0.01
Other (*n* = 67)	6.5 ± 3.8	5.3	4.7 ± 4.3	4.7	− 1.8	− 38.3%	− 2.9	< 0.01
Ethnicity								
Hispanic (*n* = 262)	6.7 ± 4.2	5.7	4.7 ± 5.1	3.6	−2.0	− 42.6%	− 6.4	< 0.01
Non-Hispanic (*n* = 374)	7.2 ± 4.6	5.5	5.2 ± 5.7	3.7	− 2.1	− 38.5%	− 7.2	< 0.01
3. Fruits and vegetables (cup equivalents/day)								
All (*n* = 647)	2.5 ± 0.9	2.3	2.1 ± 1.4	1.9	− 0.4	− 19.0%	− 7.2	< 0.01
Sex and age								
Male (*n* = 321)	2.7 ± 1.0	2.4	2.1 ± 1.4	1.8	− 0.6	−28.6%	− 7.6	< 0.01
4-8y (*n* = 136)	2.5 ± 0.8	2.4	2.0 ± 1.3	1.9	− 0.5	−25.0%	−4.0	< 0.01
9-11y (*n* = 98)	2.7 ± 1.0	2.4	2.2 ± 1.6	1.7	− 0.5	− 22.7%	− 3.2	< 0.01
12-15y (*n* = 87)	3.0 ± 1.1	2.7	2.0 ± 1.4	1.8	− 1.0	− 50.0%	−6.4	< 0.01
Female (*n* = 326)	2.3 ± 0.7	2.2	2.1 ± 1.3	1.9	− 0.2	− 9.5%	− 2.4	0.02
4-8y (*n* = 130)	2.4 ± 0.7	2.3	2.1 ± 1.3	1.8	− 0.3	− 14.3%	−2.6	0.01
9-11y (*n* = 111)	2.3 ± 0.6	2.2	2.2 ± 1.2	2.2	− 0.1	−4.5%	− 0.9	0.36
12-15y (*n* = 85)	2.2 ± 0.7	2.0	2.1 ± 1.6	1.8	− 0.1	−4.8%	− 0.6	0.55
BMI category^c^								
Normal weight (*n* = 363)	2.6 ± 0.9	2.3	2.1 ± 1.4	1.9	− 0.4	− 23.8%	− 5.8	< 0.01
Overweight (*n* = 104)	2.5 ± 0.8	2.3	2.0 ± 1.3	1.8	− 0.5	− 25.0%	− 3.5	< 0.01
Obese (*n* = 175)	2.4 ± 0.8	2.2	2.0 ± 1.3	1.8	− 0.3	− 20.0%	− 3.1	< 0.01
Race								
White (*n* = 355)	2.6 ± 0.9	2.4	2.1 ± 1.3	1.9	− 0.5	− 23.8%	− 6.5	< 0.01
African American (*n* = 134)	2.3 ± 0.7	2.1	1.9 ± 1.2	1.7	−0.4	− 21.1%	− 3.0	< 0.01
Other (*n* = 67)	2.6 ± 0.9	2.3	2.1 ± 1.4	1.9	− 0.6	− 23.8%	− 3.3	< 0.01
Ethnicity								
Hispanic (*n* = 279)	2.5 ± 0.8	2.3	2.1 ± 1.4	1.9	−0.3	− 19.0%	− 3.5	< 0.01
Non-Hispanic (*n* = 373)	2.5 ± 0.9	2.3	2.1 ± 1.3	1.8	− 0.5	−19.0%	− 6.5	< 0.01
4. Whole grains (oz equivalents/day)								
All (*n* = 652)	0.7 ± 0.4	0.6	0.5 ± 0.7	0.3	−0.2	− 40.0%	− 7.1	< 0.01
Sex and age								
Male (*n* = 322)	0.8 ± 0.5	0.7	0.5 ± 0.8	0.3	−0.2	− 60.0%	− 5.2	< 0.01
4-8y (*n* = 136)	0.8 ± 0.4	0.7	0.5 ± 0.6	0.3	−0.2	−60.0%	− 4.3	< 0.01
9-11y (*n* = 99)	0.8 ± 0.6	0.7	0.6 ± 1.0	0.3	−0.2	− 33.3%	− 2.0	0.05
12-15y (*n* = 87)	0.7 ± 0.3	0.6	0.5 ± 0.7	0.2	−0.2	− 40.0%	− 3.3	< 0.01
Female (*n* = 330)	0.6 ± 0.4	0.5	0.4 ± 0.6	0.2	− 0.2	− 50.0%	− 4.9	< 0.01
4-8y (*n* = 132)	0.6 ± 0.3	0.6	0.5 ± 0.7	0.3	−0.1	−20.0%	−1.2	0.22
9-11y (*n* = 111)	0.7 ± 0.4	0.6	0.4 ± 0.4	0.2	−0.3	−75.0%	− 5.5	< 0.01
12-15y (*n* = 87)	0.6 ± 0.5	0.5	0.4 ± 0.6	0.1	−0.2	−50.0%	−2.8	0.01
BMI category^c^								
Normal weight (*n* = 364)	0.7 ± 0.4	0.6	0.5 ± 0.7	0.3	−0.2	− 40.0%	− 5.4	< 0.01
Overweight (*n* = 105)	0.7 ± 0.4	0.6	0.5 ± 0.6	0.3	− 0.2	− 40.0%	− 3.2	< 0.01
Obese (*n* = 178)	0.7 ± 0.4	0.5	0.5 ± 0.7	0.2	− 0.2	− 40.0%	− 3.1	< 0.01
Race								
White (*n* = 358)	0.7 ± 0.4	0.6	0.5 ± 0.7	0.3	− 0.2	− 40.0%	−4.5	< 0.01
African American (*n* = 135)	0.7 ± 0.5	0.6	0.4 ± 0.5	0.2	− 0.4	− 75.0%	− 6.5	< 0.01
Other (*n* = 67)	0.7 ± 0.4	0.6	0.6 ± 0.6	0.3	−0.1	−16.7%	− 1.6	0.12
Ethnicity								
Hispanic (*n* = 264)	0.7 ± 0.4	0.6	0.5 ± 0.7	0.2	−0.2	−40.0%	−4.5	< 0.01
Non-Hispanic (*n* = 372)	0.7 ± 0.4	0.6	0.5 ± 0.6	0.3	−0.2	−40.0%	− 5.2	< 0.01
5. Fiber (g/day)								
All (*n* = 638)	15.4 ± 3.9	14.7	12.5 ± 6.5	11.4	−2.9	− 23.2%	− 10.6	< 0.01
Sex and age								
Male (*n* = 318)	16.5 ± 4.4	16.0	12.8 ± 7.2	11.7	− 3.7	−28.9%	−8.6	< 0.01
4-8y (*n* = 136)	15.4 ± 3.8	15.1	12.5 ± 6.5	12.0	−2.9	−23.2%	−4.8	< 0.01
9-11y (*n* = 99)	16.2 ± 4.7	15.6	13.7 ± 7.5	11.7	−2.5	−18.2%	−3.1	< 0.01
12-15y (*n* = 87)	18.7 ± 4.4	17.7	12.3 ± 7.8	10.7	−6.4	− 52.0%	−7.3	< 0.01
Female (*n* = 320)	14.2 ± 2.9	13.7	12.2 ± 5.8	11.3	−2.0	−16.4%	−6.4	< 0.01
4-8y (*n* = 129)	14.4 ± 2.7	14.0	11.8 ± 5.2	11.3	−2.6	− 22.0%	− 5.7	< 0.01
9-11y (*n* = 109)	14.0 ± 2.7	13.7	12.7 ± 5.8	11.3	−1.3	−10.2%	−2.5	0.02
12-15y (*n* = 82)	14.1 ± 3.3	13.3	12.1 ± 6.7	11.3	−2.0	−16.5%	−2.9	0.01
BMI category^c^								
Normal weight (*n* = 359)	15.7 ± 4.2	15.0	12.8 ± 6.8	11.7	−2.9	− 22.7%	− 7.6	< 0.01
Overweight (*n* = 103)	15.3 ± 3.8	14.8	11.8 ± 6.6	10.7	−3.6	− 29.7%	− 5.4	< 0.01
Obese (*n* = 171)	14.6 ± 3.3	14.2	12.0 ± 5.8	11.2	− 2.6	− 21.7%	−5.4	< 0.01
Race								
White (*n* = 352)	15.7 ± 4.0	15.1	12.8 ± 6.6	11.5	− 2.8	− 22.7%	− 7.9	< 0.01
African American (*n* = 131)	14.4 ± 3.9	13.4	11.3 ± 5.1	11.0	− 3.1	− 27.4%	− 5.7	< 0.01
Other (*n* = 66)	15.4 ± 3.7	14.9	12.2 ± 6.9	11.6	− 3.2	− 26.2%	− 3.7	< 0.01
Ethnicity								
Hispanic (*n* = 255)	15.5 ± 4.0	15.0	12.6 ± 7.0	11.0	−2.8	− 23.0%	− 6.2	< 0.01
Non-Hispanic (*n* = 368)	15.2 ± 3.9	14.5	12.4 ± 6.1	11.7	−2.9	− 22.6%	− 8.6	< 0.01
6. Dairy (cup equivalents/day)								
All (*n* = 653)	2.5 ± 0.7	2.4	1.8 ± 1.2	1.6	−0.7	− 38.9%	− 14.6	< 0.01
Sex and age								
Male (*n* = 323)	2.7 ± 0.7	2.7	2.0 ± 1.3	1.9	−0.7	− 35.0%	− 9.6	< 0.01
4-8y (*n* = 136)	2.8 ± 0.6	2.7	2.0 ± 1.1	1.9	−0.8	−40.0%	− 8.6	< 0.01
9-11y (*n* = 100)	2.8 ± 0.8	2.6	2.1 ± 1.3	2.1	−0.7	−33.3%	−4.6	< 0.01
12-15y (*n* = 87)	2.5 ± 0.7	2.5	2.0 ± 1.4	1.7	−0.6	− 25.0%	−3.8	< 0.01
Female (*n* = 330)	2.2 ± 0.6	2.2	1.6 ± 1.1	1.4	−0.7	− 37.5%	−11.2	< 0.01
4-8y (*n* = 131)	2.3 ± 0.5	2.2	1.7 ± 1.1	1.6	−0.6	− 35.3%	− 6.0	< 0.01
9-11y (*n* = 113)	2.5 ± 0.7	2.3	1.7 ± 1.2	1.6	−0.8	− 47.1%	− 6.6	< 0.01
12-15y (*n* = 86)	1.9 ± 0.5	1.8	1.2 ± 0.9	1.1	− 0.7	− 58.3%	− 7.2	< 0.01
BMI category^c^								
Normal weight (*n* = 364)	2.5 ± 0.7	2.4	1.8 ± 1.2	1.7	−0.7	−38.9%	− 11.3	< 0.01
Overweight (*n* = 105)	2.4 ± 0.7	2.3	1.8 ± 1.2	1.6	−0.6	− 33.3%	−5.7	< 0.01
Obese (*n* = 179)	2.5 ± 0.8	2.3	1.8 ± 1.2	1.6	−0.7	−38.9%	−7.9	< 0.01
Race								
White (*n* = 359)	2.5 ± 0.7	2.4	2.0 ± 1.2	1.9	−0.6	− 25.0%	− 8.7	< 0.01
African American (*n* = 134)	2.4 ± 0.7	2.3	1.4 ± 1.1	1.3	−0.9	−71.4%	−8.9	< 0.01
Other (*n* = 67)	2.5 ± 0.8	2.3	1.9 ± 1.3	1.8	−0.6	−31.6%	−4.5	< 0.01
Ethnicity								
Hispanic (*n* = 265)	2.5 ± 0.7	2.4	1.7 ± 1.2	1.6	−0.8	− 47.1%	− 10.2	< 0.01
Non-Hispanic (*n* = 372)	2.5 ± 0.7	2.4	1.8 ± 1.2	1.7	−0.6	−38.9%	− 10.0	< 0.01

^a^(ASA24-Kids – DSQ)

^b^(ASA24-Kids – DSQ)/ASA24-Kids

^c^BMI categories defined as follows: ‘normal weight’ was defined as having a BMI under the 85th percentile BMI for age and sex; 85th up to 95th percentile for ‘overweight’; and at or above 95th percentile for ‘obese’

Deattenuated correlation coefficients of 0.4 or above were observed for the overall sample on three of the six nutrient/food intakes measured by DSQ and ASA24-Kids; the highest deattenuated correlation was observed for sugars from SSBs (0.54), followed by dairy (0.50), and fruits and vegetables (0.40) (Table 3). Values for the other three intake variables ranged from 0.34 for whole grains and fiber to 0.37 for total added sugar. Deattenuated correlations varied among the 14 sub-groups as defined by children’s age and sex (6 groups), race (3 groups) ethnicity (2 groups), or BMI (3 groups). The greatest number of deattenuated correlation coefficients at or above 0.4 among sub-groups were seen for added sugars from SSBs (11), dairy (11), and fruits and vegetables (9). Lowest deattenuated correlations (0.15 or below) were seen among children aged 9–11 years for several dietary intakes including total added sugars (both sexes), whole grains (girls), and dairy (boys). African American children tended to have lower deattenuated correlations as compared to White children or children of other races. Hispanic children had notably higher deattenuated correlations than non-Hispanic children for intakes of total added sugars and sugars from SSBs, but substantially lower deattenuated correlations for intakes of fruits and vegetables, and whole grains. Compared to normal weight children, deattenuated correlations were higher among overweight or obese children for intakes of dairy, added sugars from SSBs, and whole grains; and lower for intake of fruits and vegetables.

**Table 3:** Slopes^a^, deattenuated correlations^b^, and attenuation factors^c^ of DSQ dietary intakes, Healthy Communities Study, USA, 2013–2015

Food group/nutrient	Slope (SEE)	Deattenuated Correlation (SEE)	Attenuation Factor (SEE)
1. Total added sugars (tsp/day)			
All (*n* = 649)	0.55 (0.11)	0.37 (0.06)	0.25 (0.04)
Sex and age			
Male (*n* = 321)	0.49 (0.14)	0.35 (0.08)	0.25 (0.06)
4-8y (*n* = 136)	0.47 (0.27)	0.36 (0.16)	0.27 (0.12)
9-11y (*n* = 98)	0.10 (0.23)	0.06 (0.15)	0.04 (0.10)^d^
12-15y (*n* = 87)	0.65 (0.29)	0.47 (0.15)	0.34 (0.11)
Female (*n* = 328)	0.57 (0.19)	0.37 (0.10)	0.24 (0.06)
4-8y (*n* = 131)	0.78 (0.97)	0.46 (0.33)	0.27 (0.14)
9-11y (*n* = 112)	0.05 (0.12)	0.06 (0.13)	0.07 (0.16)^d^
12-15y (*n* = 85)	1.60 (0.88)	0.68 (0.21)	0.29 (0.08)
BMI category^e^			
Normal weight (*n* = 363)	0.55 (0.15)	0.36 (0.08)	0.23 (0.05)
Overweight (*n* = 104)	0.39 (0.37)	0.26 (0.20)	0.18 (0.13)^d^
Obese (*n* = 177)	0.61 (0.19)	0.45 (0.10)	0.33 (0.08)
Race			
White (*n* = 357)	0.56 (0.15)	0.45 (0.09)	0.36 (0.07)
African American (*n* = 135)	0.36 (0.22)	0.20 (0.12)	0.12 (0.07)^d^
Other (*n* = 66)	0.28 (0.23)	0.27 (0.20)	0.26 (0.19)
Ethnicity			
Hispanic (*n* = 262)	1.01 (0.46)	0.60 (0.15)	0.36 (0.07)
Non-Hispanic (*n* = 371)	0.43 (0.11)	0.32 (0.07)	0.24 (0.05)
2. Added sugars from sugar sweetened beverages (tsp/day)			
All (*n* = 652)	0.66 (0.07)	0.54 (0.04)	0.44 (0.04)
Sex and age			
Male (*n* = 321)	0.71 (0.10)	0.76 (0.06)	0.81 (0.07)
4-8y (*n* = 136)	0.75 (0.29)	0.59 (0.15)	0.46 (0.11)
9-11y (*n* = 98)	0.72 (0.26)	0.77 (0.15)	0.83 (0.15)
12-15y (*n* = 87)	0.57 (0.13)	0.76 (0.10)	1.02 (0.16)
Female (*n* = 331)	0.60 (0.11)	0.40 (0.06)	0.27 (0.05)
4-8y (*n* = 132)	0.88 (0.56)	0.56 (0.21)	0.36 (0.11)
9-11y (*n* = 112)	0.19 (0.08)	0.27 (0.11)	0.39 (0.17)
12-15y (*n* = 87)	0.93 (0.23)	0.50 (0.10)	0.27 (0.06)
BMI category^e^			
Normal weight (*n* = 365)	0.50 (0.09)	0.45 (0.06)	0.40 (0.06)
Overweight (*n* = 104)	1.00 (0.54)	0.65 (0.20)	0.43 (0.11)
Obese (*n* = 178)	0.82 (0.13)	0.63 (0.07)	0.48 (0.06)
Race			
White (*n* = 358)	0.63 (0.08)	0.62 (0.05)	0.62 (0.06)
African American (*n* = 136)	0.69 (0.24)	0.39 (0.11)	0.22 (0.07)
Other (*n* = 67)	0.38 (0.22)	0.31 (0.16)	0.25 (0.14)
Ethnicity			
Hispanic (*n* = 262)	1.19 (0.40)	0.73 (0.13)	0.45 (0.06)
Non-Hispanic (*n* = 374)	0.57 (0.07)	0.56 (0.05)	0.55 (0.06)
3. Fruits and vegetables (cup equivalents/day)			
All (*n* = 647)	0.36 (0.06)	0.40 (0.05)	0.44 (0.06)
Sex and age			
Male (*n* = 321)	0.42 (0.10)	0.41 (0.08)	0.40 (0.08)
4-8y (*n* = 136)	0.31 (0.12)	0.36 (0.11)	0.42 (0.13)
9-11y (*n* = 98)	0.44 (0.17)	0.46 (0.13)	0.48 (0.15)
12-15y (*n* = 87)	0.59 (0.26)	0.46 (0.15)	0.36 (0.12)
Female (*n* = 326)	0.32 (0.07)	0.44 (0.07)	0.61 (0.10)
4-8y (*n* = 130)	0.39 (0.09)	0.54 (0.10)	0.75 (0.15)
9-11y (*n* = 111)	0.40 (0.18)	0.50 (0.15)	0.62 (0.18)
12-15y (*n* = 85)	0.19 (0.11)	0.28 (0.14)	0.42 (0.22)
BMI category^e^			
Normal weight (*n* = 363)	0.42 (0.07)	0.46 (0.06)	0.50 (0.07)
Overweight (*n* = 104)	0.49 (0.30)	0.42 (0.18)	0.36 (0.15)
Obese (*n* = 175)	0.20 (0.11)	0.23 (0.11)	0.26 (0.13)
Race			
White (*n* = 355)	0.46 (0.09)	0.47 (0.07)	0.49 (0.07)
African American (*n* = 134)	0.08 (0.13)	0.09 (0.14)	0.10 (0.15)^d^
Other (*n* = 67)	0.53 (0.21)	0.56 (0.16)	0.60 (0.18)
Ethnicity			
Hispanic (*n* = 279)	0.29 (0.10)	0.32 (0.09)	0.35 (0.10)
Non-Hispanic (*n* = 373)	0.44 (0.08)	0.47 (0.07)	0.51 (0.07)
4. Whole grains (oz equivalents/day)			
All (*n* = 652)	0.33 (0.07)	0.34 (0.06)	0.35 (0.06)
Sex and age			
Male (*n* = 322)	0.34 (0.10)	0.36 (0.08)	0.37 (0.09)
4-8y (*n* = 136)	0.38 (0.12)	0.42 (0.11)	0.47 (0.13)
9-11y (*n* = 99)	0.28 (0.19)	0.26 (0.15)	0.24 (0.14)
12-15y (*n* = 87)	0.40 (0.26)	0.47 (0.21)	0.56 (0.23)
Female (*n* = 330)	0.29 (0.09)	0.29 (0.08)	0.30 (0.08)
4-8y (*n* = 132)	0.14 (0.07)	0.26 (0.11)	0.47 (0.20)
9-11y (*n* = 111)	1.08 (38.71)	0.09 (1.87)	0.01 (0.10)^d^
12-15y (*n* = 87)	0.50 (0.17)	0.47 (0.13)	0.44 (0.13)
BMI category^e^			
Normal weight (*n* = 364)	0.27 (0.07)	0.30 (0.07)	0.32 (0.08)
Overweight (*n* = 105)	0.59 (0.39)	0.44 (0.20)	0.33 (0.13)
Obese (*n* = 178)	0.51 (0.19)	0.54 (0.13)	0.56 (0.12)
Race			
White (*n* = 358)	0.36 (0.10)	0.37 (0.08)	0.39 (0.08)
African American (*n* = 135)	0.55 (0.25)	0.35 (0.13)	0.22 (0.08)
Other (*n* = 67)	0.97 (0.84)	0.70 (0.33)	0.51 (0.17)
Ethnicity			
Hispanic (*n* = 264)	0.17 (0.06)	0.22 (0.07)	0.27 (0.09)
Non-Hispanic (*n* = 372)	0.53 (0.17)	0.45 (0.10)	0.38 (0.08)
5. Fiber (g/day)			
All (*n* = 638)	0.31 (0.06)	0.34 (0.06)	0.37 (0.06)
Sex and age			
Male (*n* = 318)	0.21 (0.07)	0.24 (0.08)	0.26 (0.09)
4-8y (*n* = 136)	0.22 (0.12)	0.26 (0.12)	0.29 (0.14)
9-11y (*n* = 99)	0.28 (0.15)	0.30 (0.14)	0.31 (0.15)
12-15y (*n* = 87)	0.18 (0.11)	0.24 (0.14)	0.31 (0.18)
Female (*n* = 320)	0.44 (0.11)	0.54 (0.09)	0.64 (0.11)
4-8y (*n* = 129)	0.56 (0.29)	0.56 (0.18)	0.57 (0.16)
9-11y (*n* = 109)	0.53 (0.26)	0.63 (0.18)	0.74 (0.19)
12-15y (*n* = 82)	0.35 (0.14)	0.48 (0.14)	0.67 (0.21)
BMI category^e^			
Normal weight (*n* = 359)	0.27 (0.07)	0.32 (0.07)	0.37 (0.08)
Overweight (*n* = 103)	0.42 (0.23)	0.40 (0.16)	0.39 (0.16)
Obese (*n* = 171)	0.35 (0.22)	0.32 (0.15)	0.29 (0.13)
Race			
White (*n* = 352)	0.32 (0.07)	0.36 (0.07)	0.41 (0.08)
African American (*n* = 131)	0.19 (0.24)	0.14 (0.16)	0.10 (0.11)^d^
Other (*n* = 66)	0.28 (0.17)	0.36 (0.18)	0.46 (0.23)
Ethnicity			
Hispanic (*n* = 255)	0.31 (0.10)	0.34 (0.09)	0.38 (0.10)
Non-Hispanic (*n* = 368)	0.35 (0.09)	0.36 (0.07)	0.37 (0.08)
6. Dairy (cup equivalents/day)			
All (*n* = 653)	0.47 (0.08)	0.50 (0.06)	0.54 (0.06)
Sex and age			
Male (*n* = 323)	0.36 (0.10)	0.39 (0.09)	0.43 (0.09)
4-8y (*n* = 136)	0.60 (0.22)	0.65 (0.14)	0.69 (0.14)
9-11y (*n* = 100)	0.19 (0.28)	0.15 (0.21)	0.12 (0.16)^d^
12-15y (*n* = 87)	0.26 (0.10)	0.38 (0.13)	0.54 (0.20)
Female (*n* = 330)	0.47 (0.12)	0.50 (0.09)	0.54 (0.09)
4-8y (*n* = 131)	0.34 (0.10)	0.49 (0.11)	0.71 (0.17)
9-11y (*n* = 113)	0.42 (0.25)	0.40 (0.18)	0.39 (0.16)
12-15y (*n* = 86)	0.77 (0.92)	0.51 (0.37)	0.34 (0.18)
BMI Category^e^			
Normal weight (*n* = 364)	0.42 (0.09)	0.48 (0.07)	0.54 (0.08)
Overweight (*n* = 105)	0.55 (0.31)	0.51 (0.19)	0.48 (0.17)
Obese (*n* = 179)	0.91 (0.42)	0.71 (0.18)	0.56 (0.11)
Race			
White (*n* = 359)	0.52 (0.13)	0.54 (0.09)	0.55 (0.09)
African American (*n* = 134)	0.22 (0.12)	0.25 (0.12)	0.27 (0.14)
Other (*n* = 67)	0.86 (0.34)	0.84 (0.17)	0.81 (0.15)
Ethnicity			
Hispanic (*n* = 265)	0.56 (0.17)	0.52 (0.11)	0.49 (0.09)
Non-Hispanic (*n* = 372)	0.42 (0.08)	0.49 (0.07)	0.57 (0.09)

^a^Slopes in the regression of dietary intake reported from Dietary Screener Questionnaire on true usual intake, as estimated from multiple ASA24-Kids recalls in a measurement error model. If the DSQ were unbiased, slopes would equal 1 [23]

^b^Deattenuated correlations between reported and true usual intakes

^c^Attenuation factors resulting from measurement error in reported intake for each dietary factor. The attenuation factor indicates the degree to which the observed log relative risk between the observed exposure of a dietary intake derived from the DSQ and an outcome of interest is biased due to measurement error [23]. Values between 0 and 1 indicate bias towards the null (underestimation of true association) and values greater than 1 indicate bias away from the null (overestimation of true association) [23]

^d^Attenuation factor is very close to 0; careful consideration is needed when determining whether to adjust these intake estimates for measurement error

^e^BMI categories defined as follows: ‘normal weight’ was defined as having a BMI under the 85th percentile BMI for age and sex; 85th up to 95th percentile for ‘overweight’; and at or above 95th percentile for ‘obese’

Attenuation was greater than 0.2 for more than 90% of the age and sex-, race-, ethnicity-, and BMI category-specific food group/nutrient intakes (Table 3). Attenuation factors for intake of sugars from SSBs were higher, while those for fruit and vegetable intake were lower, among children with higher BMI. For all intake estimates, there was a greater degree of attenuation among African American children compared to White children and children of other races. For all intake estimates other than those for total added sugars and fiber, there was a greater degree of attenuation for Hispanics than for non-Hispanics. Attenuation was most severe (λ = 0.01–0.18) for added sugars among boys and girls 9–11 years and among overweight children; whole grain intake among females 9–11 years; and dairy intake among boys 9–11 years.

### Intake patterns

Although most of the intake estimates derived from the DSQ were significantly different from those from ASA24-Kids recalls, some patterns of intake among subgroups were similar using both methods. For total added sugars, fiber, and dairy foods, males consumed more than females on both measures (Table 2). While older males consumed more added sugars than younger males in both the DSQ and ASA24-Kids, this pattern was not seen among females. For added sugars from SSBs, males consumed more than females, and older children consumed more than younger ones on both measures. Intake patterns by sex and age group for fruits and vegetables, and for whole grains were inconsistent between the two methods. In addition, both instruments showed that, on average, obese children had greater intakes of total added sugars and added sugars from SSBs than overweight or normal weight children, and, that non-Hispanic children had higher consumption of these than Hispanic children. Normal weight children had greater intake of fruits and vegetables than overweight or obese children as measured by both instruments.

## Discussion

This is the first study we are aware of which evaluates the performance of the DSQ among children. Unlike other dietary screeners which only yield consumption frequency, the DSQ yields quantified intake estimates based on reported frequencies of consumption. For three dietary outcomes examined in the HCS, added sugars from SSBs; fruits and vegetables; and dairy foods, deattenuated correlations were 0.4 or greater. This is an important finding for two reasons: i) many community policies and programs for obesity prevention target these foods, and ii) the level of agreement between the two dietary assessment methods suggests that the DSQ appears to be an acceptable tool for measuring these dietary outcomes for purposes of the analysis of the association between community programs and policies and dietary outcomes in studies such as the HCS and potentially for similar study populations and research questions.

The lower deattenuated correlations observed for total added sugars, compared with those for added sugars from SSBs may reflect the greater ease with which respondents can report beverage frequency than they can in reporting a wider range of foods they consume containing added sugars. Some research also suggests that foods consumed very frequently and foods consumed rarely, characteristic of many beverages, are more consistently reported than are foods that are consumed moderately frequently [24].

The variable levels of agreement seen between the two assessment methods for different age groups, in part, may be a function of the accuracy with which the selected primary respondents in the study are able to report dietary intakes. The higher correlations, suggestive of less measurement error, observed among children aged 12–15 years on several dietary outcomes may indicate that these youth were cognitively mature enough to serve as their own primary respondents. Reasonable agreement between both methods was also seen among children aged 4–8 years for several dietary outcomes, suggesting that caregiver adults were an adequate proxy reporter for this group. The higher degree of measurement error among children aged 9–11 years, especially males, as indicated by lower attenuation factors and deattenuated correlations for several dietary outcomes, suggests that they may have had difficulty in reporting their food and beverage intakes accurately, even with the assistance of a parent, findings which are consistent with those of numerous other studies [6, 25, 26].

There is substantial evidence that higher BMI is associated with higher measurement error in reporting diet, particularly underreporting of total energy intake [26–30]. Thus, it might be expected that overweight or obese children would underreport sugars from SSBs and other foods high in energy. However, relatively little is known about differential bias in reporting particular food groups among adults or children by BMI status, or the tendency for more biased reporting by overweight or obese respondents using different dietary assessment methods. Surprisingly, our study found that intake estimates of sugars from SSBs were less attenuated and had greater deattenuated correlation coefficients among overweight and obese children, indicating closer agreement between the two methods for overweight or obese children than for other children. By contrast, the degree of attenuation/measurement error in estimates of fruit and vegetable intake was higher in successively higher BMI categories. Further research is needed to identify the effect of BMI status, and hence misreporting bias on estimated intakes of selected *food groups* (beyond its effect on the accuracy of reporting energy intake).

We found that race and ethnicity were associated with the level of agreement between the two dietary assessment methods. Hispanic and African American children showed a higher degree of measurement error than children of other races and ethnicities. Previous validity studies have shown mixed results regarding performance of various dietary assessment methods among Hispanics compared with non-Hispanics; some studies found no significant differences in measures of agreement [23, 31–33], while others have found differences [1, 25, 34]. While efforts were made to design the DSQ to accommodate various Hispanic and Asian subgroups, it may not have included sufficient number or variety of important ethnic-specific foods. Alternatively, the scoring algorithms did not take into account the relative portion sizes of food items within a food group typical of Hispanic groups. Tucker et al. found that among Hispanic (Puerto Rican) adults, there was a tendency to consume larger portions of particular fruits and smaller portions of particular vegetables than the general U.S. population, comparing estimates from the 1982–1984 Hispanic Health and Nutrition Examination Survey with NHANES [34]. It is also possible that Hispanic respondents may have had more difficulty navigating the online ASA24-Kids recall due to language difficulty, or less familiarity with computers than adult and child respondents of other ethnicities. Although the Spanish language version of ASA24-Kids was offered, it was not often selected by respondents. Similar to previous studies comparing methods between African Americans and other races, we found that there was more measurement error in intake estimates among African Americans [35–37]. This may be due to similar factors to those we suggested for Hispanics (differences in foods consumed, and in portions of particular types of foods).

A limitation of our study was the uncertain validity of our reference method. For practical reasons, the ASA24-Kids was the best available method for collecting detailed dietary intake data in the HCS against which to compare data from the DSQ. The assumptions that 24-hour recalls provide unbiased estimates of usual food intake, and, further, that the ASA24-Kids, as a self-administered method, provides no additional bias over a standard interviewer-administered 24-hour recall are relatively unexplored assumptions. The use of an imperfect reference method, the ASA24-Kids recalls, may have led to inflated estimates of deattenuated correlation coefficients if the error within recall intake estimates was correlated with the error present in DSQ intake estimates.

With regards to the first assumption, it is possible that the ASA24-Kids recall method underestimated dietary intakes. We found that almost all of the intake estimates from the HCS recalls were significantly lower than those from the DSQ. In addition, the majority of mean nutrient/food intakes from the ASA24-Kids were lower than the NHANES 2009–2010 age and sex specific means, but most means were within 20% (see Additional file 2). It is widely known that 24-hour recalls underestimate energy intake [27, 38–41], but the presence and amount of reporting bias for food groups and many nutrients are currently unknown, in part due to the lack of a true gold standard. Other possible reasons for the lower estimates of dietary intakes from the ASA24-Kids relate to differences in the time periods covered by the two methods and the different types of memory needed for each method. Responses to the DSQ rely on participants’ generic memory, which provides general information about usual intake over the reference time period. By contrast, 24-hour recalls rely on specific shorter term memory to provide detailed information about particular intakes and behaviors [42].

The second assumption, that the ASA24-Kids as a self-administered method produces no additional bias over an interviewer-administered 24-hour recall, may be of concern. Unlike dietary interviewers, study children are not trained to navigate the software and identify foods that best match their intakes. One study showed that when children between the ages of 9 and 11 years reported their own dietary intakes over the past 24 hours, the ASA24-Kids was less accurate than the interviewer-administered AMPM [6]. However, other studies have shown that among computer-literate English-speaking adults, the ASA24 performed similarly to the AMPM [7, 8]. In this study, parent or adult proxies were available to assist children aged 9–11 years, but were instructed by the FDC to provide assistance *only* if asked by the child. More consistent and active assistance from parents about the foods eaten at home and portion sizes may be needed to improve accuracy. However, the lack of an in-person interviewer in the ASA24-Kids may also confer some advantages such as a possible reduction in social desirability bias. Smith et al. found that, among fourth-grade children, reporting accuracy decreased with social desirability when 24-hour recalls were administered in person by interviewers [43].

There are many methods used to compare performance of and agreement between dietary assessment methods. A key strength of this study was the use of measurement error modelling utilizing structural equation modeling to remove the within-person variation in food/nutrient intakes from recalls to estimate usual intake. This method made the estimates derived from the DSQ, designed to measure *usual* intake over a month, and those derived from up to two days of 24-hour recalls more comparable to each other. From this modelling approach, we then derived deattenuated correlations to examine the agreement between reported intake and estimated usual intake as they are a more useful and rigorous assessment than the traditional Spearman or Pearson correlation coefficients and kappa statistics, which do not adjust for intra-individual variation. The derived attenuation factors, which express the amount of potential bias due to measurement error in an exposure-outcome relationship, were also useful in assessing which dietary outcome variables would be candidates for correction of measurement error. We observed a wide range of attenuation factors, λ = 0.01–1.02. While it is true that as the attenuation factor gets further from 1 it becomes increasingly important to adjust measures of association between observed dietary intakes and outcomes of interest for measurement error, as the attenuation factor gets closer to zero, adjusted estimates become overinflated, making adjusting for error less desirable. Thus, among children 9–11 years old, intakes of total added sugars (among both sexes), whole grains (among girls), and dairy (among boys) may not be good candidates for measurement error correction. Similarly, it may not be advisable to correct for measurement error for intakes of total added sugars among overweight children, or for total added sugars, fruits and vegetables, and fiber among African American children.

## Conclusions

The importance of understanding the extent of measurement error in the dietary intake estimates used in the HCS, as measured by the DSQ, is fundamental to interpreting the results of associations between community policies and programs and child dietary outcomes. This study found higher deattenuated correlations and attenuation factors, overall, for intakes estimates of sugars from SSBs, dairy, and fruits and vegetables (foods often targeted in obesity prevention community policies and programs). Based on comparison with intake estimates derived from up to two ASA24-Kids recalls, our findings suggest that the DSQ may be an acceptable tool to measure key obesity-related dietary behaviors of interest among children for the purposes of studies like the HCS. However, further study exploring the assumptions made in using the ASA24-Kids as a reference method is warranted. In addition, our results suggest that measurement error modelling and regression calibration are viable methods for future studies to correct for measurement error within the DSQ for most of the food groups/nutrients among most of the strata examined in this study, which would otherwise lead to underestimating the magnitude of associations between nutrition exposures and outcomes of interest.

## Additional files

Additional file 1: **Table S1.** Definitions of dietary intake variables of interest by assessment method, Healthy Communities Study, USA, 2013–2015. (DOCX 12 kb)

Additional file 2: **Table S2.** Percent difference between mean intakes for dietary intakes as estimated by the Automated Self-Administered 24 Hour Recall for Children (ASA24-Kids) and Dietary Screener Questionnaire (DSQ) in the Healthy Communities Study (HCS), (*n* = 656) USA, 2013–2015, and as estimated by the National Health and Nutrition Examination Survey (NHANES), (*n* = 2166) USA, 2009–2010, by sex and age group. (DOCX 17 kb)

## Abbreviations

ASA24: Automated Self-Administered 24-hour Recall
ASA24-Kids: Automated Self-Administered 24-Hour Recalls for Children
DSQ: Dietary screener questionnaire
FDC: Field data collector
HCS: Healthy Communities Study
NCI: National Cancer Institute
NHANES: National Health and Nutrition Examination Survey
SSBs: Sugar-sweetened beverages
