# Supplementary material for: Comparison of the NHANES dietary screener questionnaire to the Automated Self-Administered 24-Hour Recall for Children in the Healthy Communities Study
Source: Nutr J. 2018 Nov 27;17:111. doi: 10.1186/s12937-018-0415-1 (PMC6260716; doi:10.1186/s12937-018-0415-1)
Supplement: Supplementary file 1 — Table S1. Definitions of dietary intake variables of interest by assessment method, Healthy Communities Study, USA, 2013–2015. (DOCX 12 kb) [file 12937_2018_415_MOESM1_ESM.docx]

**Comparison of the NHANES Dietary Screener Questionnaire to the Automated Self-Administered 24-Hour Recall for Children in the Healthy Communities Study.**

Supplementary Table 1. Definitions of dietary intake variables of interest by assessment method, Healthy Communities Study, USA, 2013-2015.

| **Dietary Intake of Interest** | **Dietary Screener Questionnaire** | **Automated Self-Administered 24 Hour Recall for Children** |
| --- | --- | --- |
| Total added sugars | Tsp/day of added sugars from: cereals, soda, sweetened coffee/tea, sports/energy drinks, sweetened fruit drinks, chocolate/candy, doughnuts, cookies/cakes/pies/brownies, and ice cream (9 questions) | Tsp/day of added sugars |
| Added sugars from sugar sweetened beverage intake | Tsp/day of added sugars from SSBs including: soda, sweetened coffee/tea, sports/energy drinks, and sweetened fruit drinks (4 questions) | Tsp/day of added sugars from SSBs |
| Fruit and vegetable intake | Cup equivalents/day of fruits and vegetables including: 100% fruit juices, fruit, leafy greens, fried potatoes, other potatoes, dried beans, other vegetables, pizza, salsa, and tomato sauces (10 questions) | Cup equivalents/day of total fruits and vegetables |
| Whole grain intake | Oz equivalents/day of whole grains from: cereals, whole grain bread, brown rice and whole grains, and popcorn (4 questions) | Oz equivalents/day of whole grains |
| Fiber intake | Grams/day of fiber from: cereals, milk, soda, 100% fruit juices, sweetened coffee/tea, sports/energy drinks, sweetened fruit drinks, fruit, leafy greens, fried potatoes, other potatoes, dried beans, other vegetables, pizza, salsa, tomato sauces, cheese, whole grain bread, brown rice and whole grains, chocolate/candy, doughnuts, cookies/cakes/pies/brownies, ice cream, and popcorn (26 questions) | Grams/day of fiber |
| Milk/dairy intake | Cup equivalents/day of dairy from: milk, pizza, cheese, and ice cream (4 questions) | Cup equivalents/day of total dairy |
