# Supplementary material for: Comparison of the NHANES dietary screener questionnaire to the Automated Self-Administered 24-Hour Recall for Children in the Healthy Communities Study
Source: Nutr J. 2018 Nov 27;17:111. doi: 10.1186/s12937-018-0415-1 (PMC6260716; doi:10.1186/s12937-018-0415-1)
Supplement: Supplementary file 2 — Table S2. Percent difference between mean intakes for dietary intakes as estimated by the Automated Self-Administered 24 Hour Recall for Children (ASA24-Kids) and Dietary Screener Questionnaire (DSQ) in the Healthy Communities Study (HCS), (n = 656) USA, 2013–2015, and as estimated by the National Health and Nutrition Examination Survey (NHANES), (n = 2166) USA, 2009–2010, by sex and age group. (DOCX 17 kb) [file 12937_2018_415_MOESM2_ESM.docx]

**Comparison of the NHANES Dietary Screener Questionnaire to the Automated Self-Administered 24-Hour Recall for Children in the Healthy Communities Study.**

Supplementary Table 2. Percent difference between mean intakes for dietary intakes as estimated by the Automated Self-Administered 24 Hour Recall for Children (ASA24-Kids) and Dietary Screener Questionnaire (DSQ) in the Healthy Communities Study (HCS), (n=656) USA, 2013-2015, and as estimated by the National Health and Nutrition Examination Survey (NHANES), (n=2166) USA, 2009-2010, by sex and age group.

| Food group/nutrient | HCS Sample ASA24-Kids Mean | HCS Sample DSQ Mean | NHANES Mean | Percent Difference between ASA24-Kids and NHANES | Percent Difference between DSQ and NHANES |
| --- | --- | --- | --- | --- | --- |
| 1. Total added sugars (tsp/day) | | | | | |
| All | 12.2 | 19.0 | 16.2 | -24.9% | 17.1% |
| Male |  |  |  |  |  |
| 4-8y | 11.0 | 18.3 | 15.3 | -27.9% | 19.3% |
| 9-11y | 13.8 | 19.6 | 19.1 | -27.5% | 2.5% |
| 12-15y | 15.2 | 23.5 | 18.9 | -19.4% | 24.4% |
| Female |  |  |  |  |  |
| 4-8y | 10.6 | 16.0 | 12.9 | -18.2% | 24.2% |
| 9-11y | 12.8 | 18.2 | 16.6 | -23.1% | 9.9% |
| 12-15y | 10.6 | 20.3 | 16.2 | -34.5% | 25.5% |
| 2. Added sugars for sugar sweetened beverages (tsp/day) | | | | | |
| All | 4.9 | 7.1 | 5.8 | -15.1% | 21.9% |
| Male |  |  |  |  |  |
| 4-8y | 4.0 | 6.1 | 4.5 | -11.1% | 35.1% |
| 9-11y | 5.6 | 6.6 | 7.1 | -21.5% | -7.0% |
| 12-15y | 7.5 | 10.3 | 8.6 | -12.2% | 20.0% |
| Female |  |  |  |  |  |
| 4-8y | 3.8 | 5.3 | 3.4 | 10.4% | 55.7% |
| 9-11y | 5.1 | 6.0 | 5.6 | -8.4% | 6.5% |
| 12-15y | 4.5 | 10.0 | 6.8 | -33.4% | 47.2% |
| 3. Fruits and vegetables (cup equivalents/day) | | | | | |
| All | 2.1 | 2.5 | 2.1 | 0.2% | 19.3% |
| Male |  |  |  |  |  |
| 4-8y | 2.0 | 2.5 | 2.1 | -2.8% | 19.6% |
| 9-11y | 2.2 | 2.7 | 2.0 | 8.0% | 34.5% |
| 12-15y | 2.0 | 3.0 | 2.0 | -0.2% | 50.3% |
| Female |  |  |  |  |  |
| 4-8y | 2.1 | 2.4 | 2.2 | -2.7% | 9.7% |
| 9-11y | 2.2 | 2.3 | 2.1 | 4.4% | 9.4% |
| 12-15y | 2.1 | 2.2 | 2.1 | -0.7% | 4.3% |
| 4. Whole grains (oz equivalents/day) | | | | | |
| All | 0.5 | 0.7 | 0.7 | -29.3% | -0.6% |
| Male |  |  |  |  |  |
| 4-8y | 0.5 | 0.8 | 0.8 | -33.4% | -4.3% |
| 9-11y | 0.6 | 0.8 | 0.7 | -10.3% | 20.1% |
| 12-15y | 0.5 | 0.7 | 0.7 | -34.3% | 0.0% |
| Female |  |  |  |  |  |
| 4-8y | 0.5 | 0.6 | 0.6 | -9.9% | 3.1% |
| 9-11y | 0.4 | 0.7 | 0.6 | -37.5% | 9.3% |
| 12-15y | 0.4 | 0.6 | 0.5 | -19.5% | 17.5% |
| 5. Fiber (g/day) | | | | | |
| All | 12.5 | 15.4 | 13.6 | -8.1% | 13.0% |
| Male |  |  |  |  |  |
| 4-8y | 12.5 | 15.4 | 13.4 | -6.5% | 14.9% |
| 9-11y | 13.7 | 16.2 | 14.9 | -8.0% | 8.8% |
| 12-15y | 12.3 | 18.7 | 14.3 | -14.1% | 30.8% |
| Female |  |  |  |  |  |
| 4-8y | 11.8 | 14.4 | 12.6 | -6.2% | 14.4% |
| 9-11y | 12.7 | 14.0 | 14.0 | -9.4% | -0.2% |
| 12-15y | 12.1 | 14.1 | 13.4 | -9.7% | 5.2% |
| 6. Dairy (cup equivalents/day) | | | | | |
| All | 1.8 | 2.5 | 2.3 | -22.1% | 7.6% |
| Male |  |  |  |  |  |
| 4-8y | 2.0 | 2.8 | 2.5 | -19.7% | 10.3% |
| 9-11y | 2.1 | 2.8 | 2.5 | -14.8% | 12.4% |
| 12-15y | 2.0 | 2.5 | 2.4 | -17.9% | 6.1% |
| Female |  |  |  |  |  |
| 4-8y | 1.7 | 2.3 | 2.1 | -18.9% | 7.5% |
| 9-11y | 1.7 | 2.5 | 2.1 | -19.8% | 17.9% |
| 12-15y | 1.2 | 1.9 | 2.0 | -42.1% | -5.7% |
